# A Multiple-Medical-Image Encryption Method Based on SHA-256 and DNA Encoding

**DOI:** 10.3390/e25060898

**Published:** 2023-06-03

**Authors:** Junfeng Wu, Jialu Zhang, Dong Liu, Xiaofeng Wang

**Affiliations:** Department of Applied Mathematics, Xi’an University of Technology, Xi’an 710049, China; faziboat@126.com (J.Z.); liudong9813@163.com (D.L.); xfwang@xaut.edu.cn (X.W.)

**Keywords:** medical image encryption, multiple-image encryption, hash function, linear congruence, DNA encoding

## Abstract

Ensuring the privacy and secrecy of digital medical images has become a pressing issue as a result of the quick development of smart medical technology and the exponential growth in the quantity of medical images transmitted and stored in networks. The lightweight multiple-image encryption approach for medical images that is suggested in this research can encrypt/decrypt any number of medical photos of varied sizes with just one encryption operation and has a computational cost that is similar to encrypting a single image. The plaintext images with different sizes are filled at the right and bottom of the image to ensure that the size of all plaintext images is uniform; then, all the filled images are stacked to obtain a superimposed image. The initial key, which is generated using the SHA-256 technique, is then used as the starting value of the linear congruence algorithm to create the encryption key sequence. The cipher picture is then created by encrypting the superimposed image with the encryption key and DNA encoding. The algorithm can be made even more secure by implementing a decryption mechanism that decrypts the image independently in order to reduce the possibility of information leaking during the decryption process. The outcomes of the simulation experiment demonstrate the algorithm’s strong security and resistance to interference such as noise pollution and lost image content.

## 1. Introduction

Digital medical images are becoming increasingly important in the diagnosis and treatment of medical diseases. The era of digital medicine and telemedicine has arrived with the advancement of information technology and artificial intelligence. A large number of medical images are created using various imaging technologies (such as X-ray CT, MRI, etc.). These medical images are transmitted and stored over the network for medical diagnosis, disease monitoring, and other purposes. However, the public transmission of medical images on the internet would expose patients’ privacy, so how to protect the security of medical images has become a major challenge [1,2].

Medical image encryption is an effective method for protecting medical images from unauthorized access and ensuring a secure connection. Several medical image encryption methods have been proposed in recent years. For example, Pareek, N. K. et al. [3] proposed a healthcare image encryption algorithm based on confusion and diffusion operations performed with Arnold’s transformation, a logistic chart, and a Henon map. Cao W J et al. [4] created a partial encryption scheme for medical image edge maps that employs XOR, random bit sequence generation, bit plane transformation, and other components to form a medical image encryption algorithm. Dolendro SL et al. [5] presented a medical image encryption scheme using an improved ElGamal encryption technique. Hua Z.Y. et al. [6] suggested a high-speed scrambling and pixel adaptive diffusion scheme to protect medical images, which is composed of random data insertion, high-speed scrambling, and pixel adaptive diffusion. Liu J Z et al. [7] investigated a scheme based on a simple chaotic system that utilizes a hyperbolic sine for medical image encryption. Hossein N et al. [8] offered a hybrid model of the modified genetic algorithm and coupled map lattices. Chai X L et al. [9] combined a Latin square and chaotic system for medical image encryption. Jain, K et al. [10] incorporated Arnold’s Cat Map and a 2D Logistic-Sine-Coupling Map for increased randomness and security of the encrypted medical image.

Recently, Yasseret I. et al. [11] explored innovative perturbation algorithms that utilize novel chaotic maps. The proposed perturbation-based data encryption is employed in both rounds of confusion and diffusion to cope with the drawbacks of traditional chaos-based confusion and diffusion architecture. Zhang Y Z et al. [12] proposed an efficient multi-level encryption scheme for stereoscopic medical images based on coupled chaotic systems and Otsu threshold segmentation. Ding Y et al. [13] introduced a deep learning-based stream cipher generator for medical image encryption and decryption. To achieve high security, Amit Kumar Sing P [14] encrypted the segmented part of a plaintext image by using a key generated by redundant discrete wavelet transform (RDWT) and randomized singular value decomposition (RSVD) and scrambled segmented images. Huang et al. [15] investigated a privacy-preserving deep neural network with learnable image encryption on medical images. Kiran et al. [16] proposed a scheme that uses the Laplacian edge detection method to segment original medical images into region of interest(ROI) and region of background parts. The ROI part is permuted using an Arnold map along with a circular method and diffused using a Duffing chaotic system with pre-defined threshold values. Siju et al. [17] proposed a hybrid chaotic model of the 2D Lorenzt chaotic model and the logistic chaotic model for the encryption/decryption of medical DICOM CT images. Subsequently, Siju et al. [18] applied a linear feedback shift register (LFSR) for IoT-based medical image encryption. Wu et al. [19] introduced a novel cryptosystem for secure healthcare with a random DNA encoding module and a content-aware permutation and diffusion module. Trujillo-Toledo et al. [20] presented real-time medical image encryption using improved sequences from chaotic maps.

Due to the limitations of hardware storage space and computational power, as well as the real-time needs of picture encryption/decryption, it is imperative that the encryption/decryption of many medical images can be accomplished with a single calculation. Multiple studies have been conducted on multi-image encryption. For example, Li CL [21] investigated a multiple-image encryption technique based on a robust chaotic map in the wavelet domain, unified permutation, and separate diffusion. Zhang XQ et al. [22] presented a multiple-picture encryption algorithm based on DNA coding, which combines numerous photos into one huge image and encrypts it with a standard image encryption algorithm. Multiple images can be encrypted with this method using a single encryption computation, but the cost of the calculation is nearly identical to that of encrypting multiple images. Zhang XQ et al. [23] suggested the 3D scrambling model and dynamic DNA coding for medical image encryption. For grayscale images, M. Demirta et al. [24] proposed a safe multiple-image encryption approach based on 3D bit-scrambling and diffusion processes. Su YF et al. [25] developed a modified iterative phase retrieval technique with a structured phase mask in the Fresnel domain. However, nearly all algorithms use conventional grayscale images as test cases, and there are virtually no encryption systems for medical images. Gao X Y et al. [26] presented a multiple-image encryption algorithm based on a 3D cube and hyper-chaotic map. Wang Y F et al. [27] proposed multiple-color-image encryption based on cascaded quaternion gyrator transforms. V. Sangavi et al. [28] introduced an exquisite multiple-image encryption harnessing multi-scroll Lu-Chen and Chua chaotic system employing the domino strategy. Shazia S et al. [29] designed a novel multiple-image encryption technique utilizing the Affine Hill cipher and reality-preserving two-dimensional discrete fractional Hartley transform and a generalized two-dimensional Arnold map. Li F G et al. [30] proposed multiple-image encryption using OAM multiplexing holography based on phase jump gradient factors.

Existing image encryption technology typically encrypts the image’s content with a single encryption operation, which is adequate for the majority of applications. In medical applications, however, a large number of confidential medical photos must be stored for an extended length of time and can be successfully sent on Internet of Things devices with limited memory and processing power. In this circumstance, standard image encryption methods are too heavy. Since only one picture may be encrypted or decrypted at a time, it is difficult for devices with limited memory to perform batch processing in a timely manner. As a result, the one-time image encryption method is incompatible with the application environment of medical image applications, which requires massive information storage and real-time transmission, and a specialized image encryption algorithm must be developed to achieve simultaneous encryption/decryption of batch images.

We propose a lightweight multi-medical-image encryption method to address the issues of efficiency and computational complexity in multiple-medical-image encryption. To meet the requirements of timeliness and real-time transmission of medical images, the proposed method makes a tradeoff between security and computing efficiency. The following are the major contributions of this research. (1) We propose a multi-image encryption method for medical images that can encrypt any number of images of varying sizes with a single encryption operation. It has the same computational cost as encrypting an image. The decryption process can perform single-image decryption, which eliminates the risk of information leakage during the decryption process.(2) The procedure creates an initial key using a hash function, and then creates an encryption key sequence using a linear congruence algorithm with the initial value of the key. It achieves multiple-image encryption using DNA coding and an encryption key that has the properties of being lightweight. (3) The image encryption/decryption technique is a lossless scheme, meaning that there will be no pixel loss throughout the encryption/decryption process and the quality of the decrypted image will be the same as the quality of the original image.

The remainder of this paper is organized as follows. The proposed multiple-medical-image encryption is explained in Section 2. Section 3 and Section 4 present the simulation results and security analysis, respectively. The paper is concluded in Section 5.

## 2. The Proposed Medical Image Encryption Scheme

**Figure 1:**
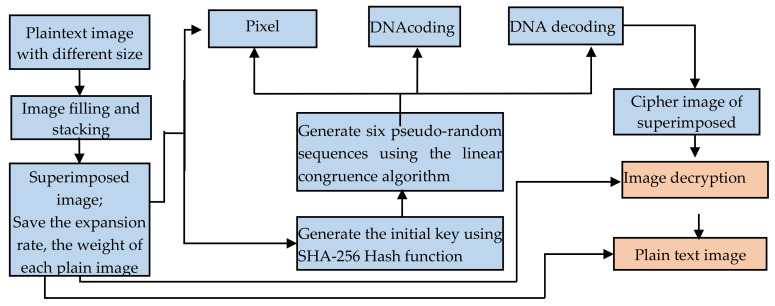
Structural flow of the encryption process.

We present a multiple-medical-image approach that encrypts several images using a single encryption computation, taking into account the hardware storage and computing restrictions in medical applications as well as the real-time needs of medical image applications. The four stages of the proposed method—image stacking, key generation, image encryption, and image decryption—are described in detail in this section. Figure 1 depicts the structure and flow of the encryption procedure.

### 2.1. Image Filling and Stacking

Based on the image stacking algorithm in our previous work [31], for medical images $P_{1},P_{2},\ldots ,P_{k}$ of any size, assume that their sizes are $M_{i}\times N_{i}(i=1,2,\ldots ,k)$, respectively. Calculate the maximum height and maximum width:

(1) $$M_{0}=max(M_{1},M_{2},\ldots ,M_{k}),\;N_{0}=max(N_{0},N_{1},\ldots ,N_{k}),$$

where $M_{0}$ and $N_{0}$ represent the maximum height and maximum width of *k* medical images, respectively. If the size of the image is not equal to $M_{0}\times N_{0}$, fill in zeros at the right and bottom of the image to make the size of the image equal to $M_{0}\times N_{0}$,which is:

(2) $$P_{i}^{\prime }=F(P_{i}),$$

where F(⋅) is the fill function. Note that the expanded images are $P_{1}^{\prime },P_{2}^{\prime },\ldots ,P_{k}^{\prime }$. In order to restore the expanded image to its original size during decryption, we define and save the expansion rate of each image, as shown in Equation (3).

(3) $$E_{i}=[M_{i}/M_{0},N_{i}/N_{0}],\;\;(i=1,2,\ldots ,k),$$

For the expanded images $P_{1}^{\prime },P_{2}^{\prime },\ldots ,P_{k}^{\prime }$, use Equation (4) to stack to form a superimposed image *P* with the same size of $M_{0}\times N_{0}$:

(4) $$P_{1}^{\prime }+P_{2}^{\prime }+\ldots +P_{k}^{\prime }=P.$$

In order to separate stacked images and restore plain images during decryption, it is necessary to record the weight of each plain image $P_{i}(i=1,2,\ldots ,k)$ pixel in the stacked image *P*, and record the weight matrix as $W_{i}(i=1,2,\ldots ,k)$. The definition of the weight matrix is as follows:

(5) $$W_{i}=P_{i}/P,\;\;(i=1,2,\ldots ,k)$$

### 2.2. Key Generation

(1) Initial key generation

Considering the security and efficiency of the cryptographic Hash function, we apply the SHA-256 function to calculate the message digest of *P*:

*K* = SHA-256(*P*). (6)

*K* is divided into 64 groups according to the order of every 4 bits, and the decimal representation of each group is calculated, which is expressed as 64 decimal values and denoted as $K_{j}(j=1,2,\ldots ,64)$. Then, divide the 64 values into 8 groups, and each group of 8 numbers will generate the initial key ${\mathrm{seed}}_{1},{\mathrm{seed}}_{2},{\mathrm{seed}}_{3},\ldots ,{\mathrm{seed}}_{8}$, as shown in Equation (7):

(7) $${\mathrm{seed}}_{m}=\sum_{j=1+(m-1)\times 8}^{8\times m}K_{i}\;,\;(m=1,2,\ldots ,8),$$

where $seed=\left\{{\mathrm{seed}}_{m}\right\}(m=1,2,\ldots ,8)$, and *seed* is the initial key.

(2) Encryption key generation

The linear congruence method is one of the most widely used pseudo-random number generation algorithms. Because of its high computational performance, it is suitable for image encryption key generation. Choose six of the eight initial keys generated in Equation (7). It is better to set $seed_{m}(m=1,2,\ldots ,6)$ as the initial value of the linear congruence algorithm. Let $X_{m}=seed_{m},(m=1,2,\ldots ,6)$, and use Equation (8) to perform the iterative operation on $X_{m}$.

(8) $$\left\{\begin{array}{c} x_{1}=(aX_{m}+c)(modT)\\ x_{2}=(ax_{1}+c)(modT)\\ \cdots \\ x_{n}=(ax_{1}+c)(modT) \end{array}\right.,\;(m=1,2,\ldots ,6),$$

where *a*, *c* are constants, *T* is the period, and *n* is the number of iterations.

### 2.3. Image Encryption

(1) Pixel scrambling

Arrange the pixels of overlay *P* as a one-dimensional array with the size of $M_{0}\times N_{0}$ For simplicity, it is still marked as *P*. Using the linear congruence algorithm (Equation (8)), take $X_{1}$ as the initial value and iterate $M_{0}\times N_{0}$ times to generate pseudo-random sequence $S_{1}$:

(9) $$S_{1}=(x_{1},x_{2},\ldots ,x_{M_{0}\times N_{0}}),$$

*P* is reordered using $S_{1}$, that is, the first scrambling of the superposition graph *P*. The sequence $S_{1}$ is sorted in order from small to large, and an ordered sequence $O\_S_{1}$ and its corresponding index sequence $idxS_{1}$ are obtained, as shown in Equation (10):

(10) $$[O\_S_{1},idxS_{1}]=sort(S_{1}),$$

where sort(⋅) represents the sort function from small to large, and the index sequence $idxS_{1}$ is used to scramble *P*. The scrambling rule is shown in Equation (11), and the generated new sequence is marked as $P^{\left(1\right)}$:

(11) $$P^{\left(1\right)}=P(idxS_{1}),$$

The sequence $P^{\left(1\right)}$ after the first scrambling can be expressed as:

(12) $$P^{\left(1\right)}=[P_{1}^{\left(1\right)},P_{2}^{\left(1\right)},\ldots ,P_{M_{0}\times N_{0}}^{\left(1\right)}],$$

Using the linear congruence algorithm (Equation (8)), a pseudo-random sequence $S_{2}$ with a length of $M_{0}\times N_{0}$ is generated with $X_{2}$ as the initial value, and the above $P^{\left(1\right)}$ is rearranged; that is, the overlay *P* is scrambled for a second time. The specific process is shown in Equation (13):

(13) $$P^{\left(2\right)}=P^{\left(1\right)}(idxS_{2}(i)),$$

where $idxS_{2}(i)$ is the index value of sequence $S_{2}$.

(2) DNA coding

DNA sequences consists of four nucleic acid bases, i.e., adenine (A), guanine (G), cytosine (C),and thymine (T), where A, T and C, G are complementary pairs. In a binary system, all digits can be represented by 0 and 1. Here, 0 and 1 are complementary; thus, 00 and 11 are complementary, and 01 and 10 are complementary. Let 00 represent adenine A, 01 represent cytosine C, 10 represent guanine G, and 11 represent thymine T; then, each 8-bit binary number can be represented by A, C, G, and T. There are 24 encoding schemes for A, C, G, and T with 00, 01, 10, and 11, but only 8 encoding schemes can satisfy the complementary rule. According to Refs. [32,33,34,35], these rules are shown in Table 1.

For a gray image, each pixel can be represented by a DNA sequence whose length is 4. For example, if the pixel value is 40, its binary value is “00 10 10 00”, which can be encoded into the DNA sequence “AGGA” using rule 1 in Table 1. Decoding is the inverse process of encoding.

DNA operation involves three algebra operations: addition, subtraction, and XOR [32,33,34,35]. Eight kinds of DNA encoding rules correspond to eight DNA operation rules. For example, for the encoding rule 1 in Table 1, the operation rules for addition, subtraction, and XOR are shown in Table 2, Table 3 and Table 4. As we can see, the DNA subtraction is the inverse operation of DNA addition. For instance, if C + G = T, then C = T − G, G = T − C. In addition, the XOR operation has good properties. That is, if T ⊕ C = G, then C = T ⊕ G, G = T ⊕ C, where ⊕ represents the XOR operation.

Take *X*_3_ and *X*_4_ as the initial values and generate pseudo-random sequences $S_{3}$ and $S_{4}$ with the length of $M_{0}\times N_{0}$, respectively, using Equation (8). Then, transform each element in sequences $S_{3}$ and $S_{4}$ according to Equation (14), map them to the interval [1,8], and form new sequences $R_{1}$ and $R_{2}$:

(14) $$\begin{array}{c} R_{1}(i)=S_{3}(i)mod(8),R_{1}=[R_{1}(1),R_{1}(2),\ldots ,R_{1}(M_{0}\times N_{0})]\\ R_{2}(i)=S_{4}(i)mod(8)+1,R_{2}=[R_{2}(1),R_{2}(2),\ldots ,R_{2}(M_{0}\times N_{0})] \end{array},\;(i=1,2,\ldots ,M_{0}\times N_{0})$$

$R_{1}$ is used to control the selection of DNA coding rules, $R_{2}$ is used to control the number of XOR operations, and $P^{\left(2\right)}$ is DNA coded to generate $P^{\left(3\right)}$:

(15) $$P^{\left(3\right)}=DNA(P^{\left(2\right)}),$$

where DNA(⋅) represents the DNA coding algorithm. The specific operation is described as follows: with $X_{5}$ as the initial value, generate a pseudo-random sequence $S_{5}$ with the length of $M_{0}\times N_{0}$ by using Equation (8), convert each element of the sequence into an integer within [0, 255] by modulo 256 operation, and then encode DNA to obtain a DNA sequence with the length of $M_{0}\times N_{0}$, as follows:

(16) $$S_{5-0}=DNA(S_{5}).$$

Let

(17) $$P^{\left(4\right)}=P^{\left(3\right)}⊕S_{5-0},$$

where ⊕ represents the XOR operation.

(3) DNA decoding

Using $X_{6}$ as the initial value, a pseudo-random sequence $S_{6}$ with a length of $M_{0}\times N_{0}$ is generated. Using the pseudo-random sequence $S_{6}$, DNA decoding is performed on the matrix obtained after the above operations to obtain the encryption diagram *Q*.

(18) $$Q=Decode(P^{\left(4\right)},S_{6}),$$

where Decode(⋅) represents the DNA decoding operation.

### 2.4. Image Decryption

In order to decrypt each image separately, the decryption key of each image $P_{i}$ is saved in the encryption process, including three parts $\left\{K,E_{i},W_{i}\right\}$, as shown in Equation (19):

(19) $$Key_{i}=\{K,E_{i},W_{i}\},$$

where *K* is $S_{1},S_{2},\ldots ,S_{6}$, $E_{i}=[M_{i}/M_{0},N_{i}/N_{0}]$, and $W_{i}=P_{i}/P,\;(i=1,2,\ldots ,k)$.

Decryption is the inverse process of encryption. The stacked image P is obtained through the inverse operation of the encryption process, and then the image *P_i_* is recovered using $\left\{E_{i},W_{i}\right\}$.

## 3. Encryption and Decryption Simulation Results

**Figure 2:**
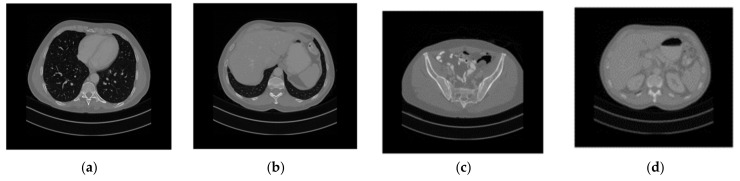
Plain medical CT images. (**a**) Original “001” image, (**b**) original “002” image, (**c**) original “003” image, (**d**) original “004” image.

In this section, we evaluate the security and efficiency of the proposed encryption algorithm. All tests were carried out on a personal computer with Intel Core i7-10750H CPU @ 2.60 GHz and 16 GB RAM, using MATLAB R2021a. The medical images used in this paper were obtained from the TCGA database (cancerimagingarchive.net). We selected four types of medical images, showing the chest, liver, pelvis, and kidney, and with different sizes to reflect the diversity of plaintext images, numbered as “001”, “002”, “003”, and “004”, respectively, as shown in Figure 2. The sizes of CT images of the chest, liver, pelvis, and kidney are 599 × 677, 480 × 542, 360 × 407, and 240 × 271, respectively.

**Figure 3:**
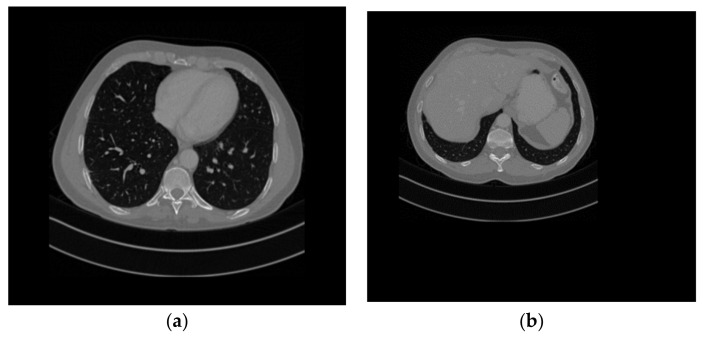

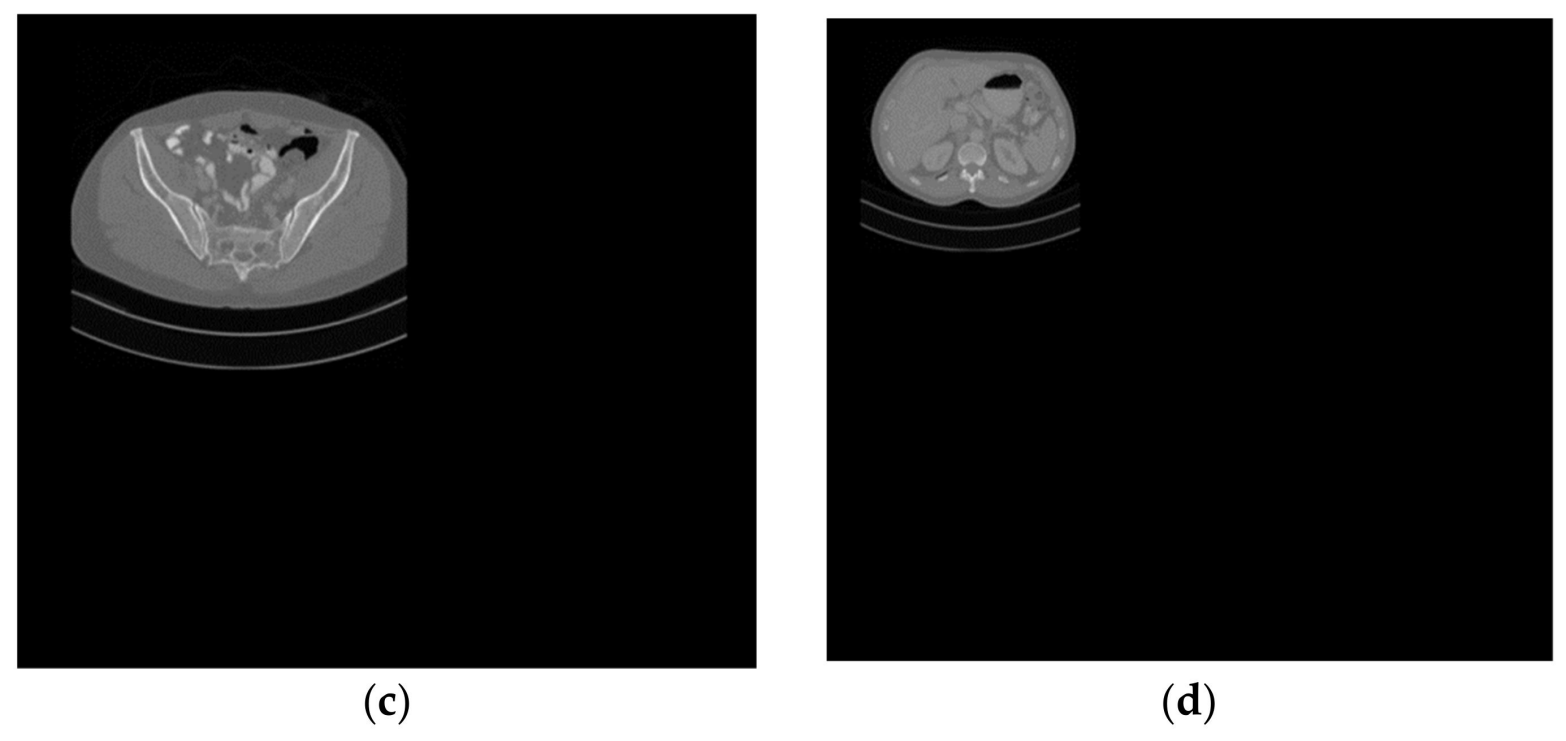
Expanded medical CT images. (**a**) Expanded “001” image, (**b**) Expanded “002” image, (**c**) Expanded “003” image, (**d**) Expanded “004” image.

**Figure 4:**
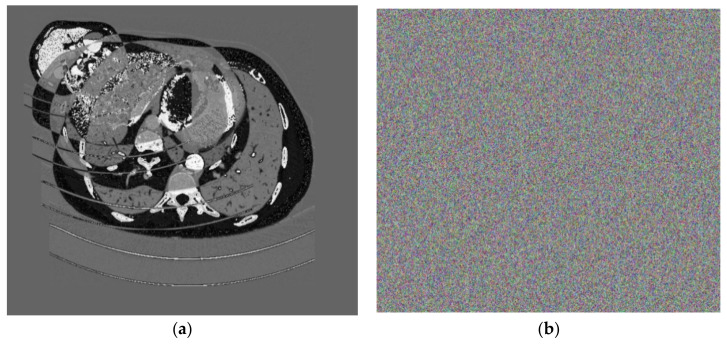
(**a**) The superimposed image, (**b**) cipher image.

**Figure 5:**
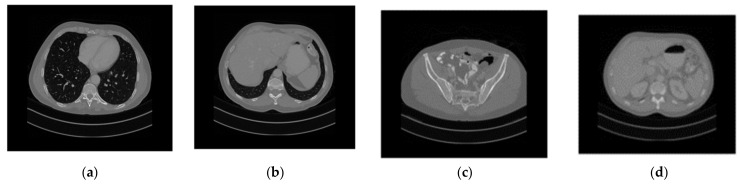
The decryption simulation results of four medical images. (**a**) Decrypted “001” image, (**b**) decrypted “002” image, (**c**) decrypted “003” image, (**d**) decrypted “004” image.

Firstly, four medical images of different sizes were expanded, and the expanded images are shown in Figure 3a–d. Then, we used the stacking algorithm described in Section 2.1 to obtain a size of 599 × 677, as shown in Figure 4a. We generated the key according to the key generation method described in Section 2.2, and encrypted the stacked image according to the encryption process described in Section 2.3. The generated encrypted image is shown in Figure 4b, and the decrypted image is shown in Figure 5.

## 4. Security Performance Analysis

### 4.1. Histogram Analysis

The statistical distribution of each pixel’s value can be seen in the image’s histogram. The distribution of the pixel value of the plain image on the histogram will typically be highly erratic and dispersed because the plain image contains a wealth of information. However, the cipher image is created from the plain image after various operations that scramble and alter it, so it no longer contains useful information. The distribution of pixel values in cipher images should follow a fairly consistent pattern in order to deter attackers from interpreting the gray distribution of pixels by looking at the histogram of encrypted images.

**Figure 6:**
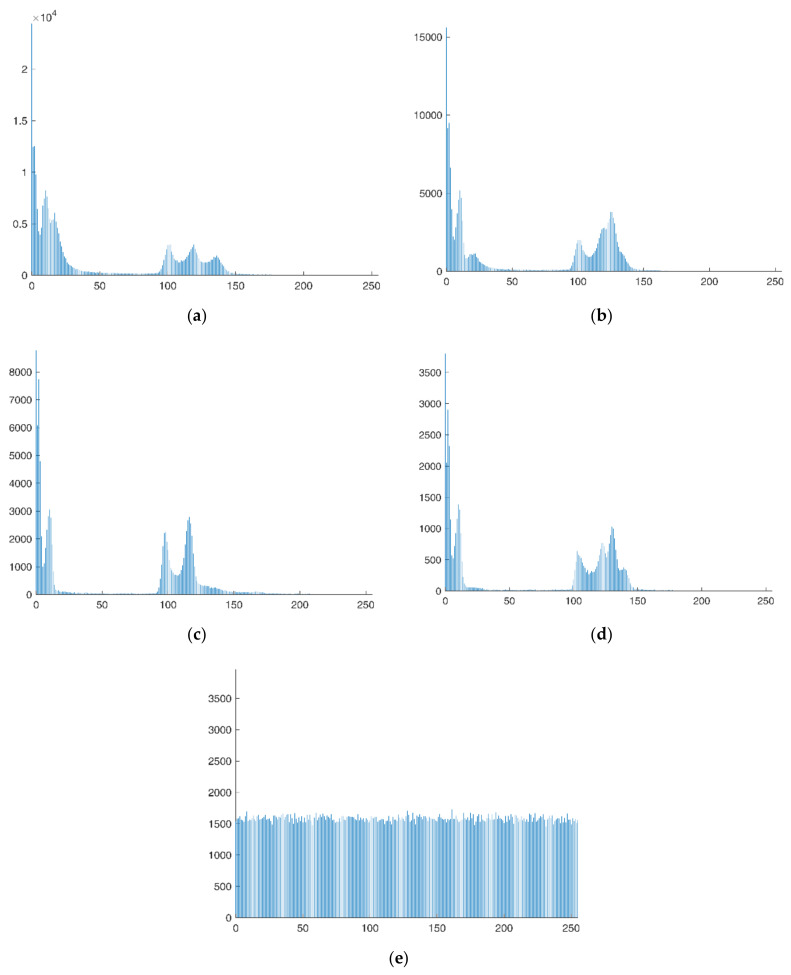
Histogram analysis results. (**a**) Histogram of “001” image, (**b**) histogram of “002” image, (**c**) histogram of “003” image, (**d**) histogram of “004” image, (**e**) histogram of encrypted image.

The best protection against a cipher attack is to have a very uniform histogram distribution of the encrypted cipher image such that the original information representing the plain image cannot be acquired by the outside world through a cipher analysis attack. Histograms of the plain image and the cipher image are displayed in Figure 6a–e. The fact that the information distribution of cipher images is greater than that of plain images shows that the algorithm can withstand attempts to decipher it using statistical analysis techniques.

### 4.2. The Correlation of Adjacent Pixels

The degree of correlation between neighboring pixels in an image is referred to as the correlation analysis of adjacent pixels. There will always be some correlation between pixels in various nearby directions of plain images because of the imaging features of plain images, where adjacent pixels’ values are typically close. The cipher image modifies the pixel value in addition to the pixel position after several scrambling and diffusion operations. As a result, there is little correlation between the pixels and it is difficult to have a correlation. A safe image encryption algorithm must be able to decrease the correlation between adjacent pixels of the cipher image to the point where it is weak or even irrelevant in order to resist statistical examination.

**Figure 7:**
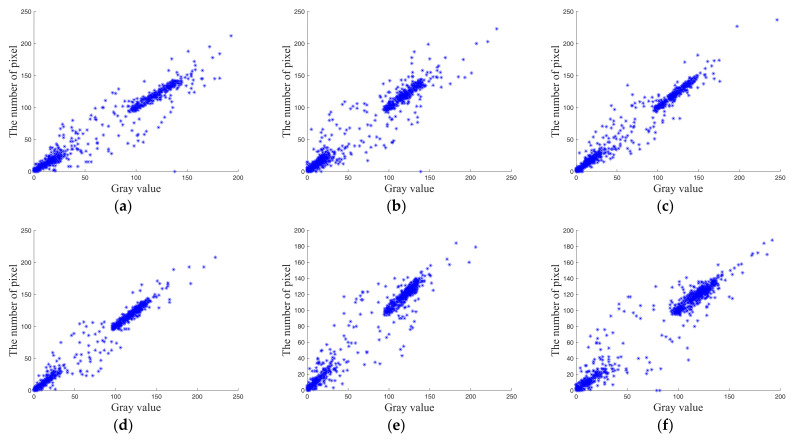

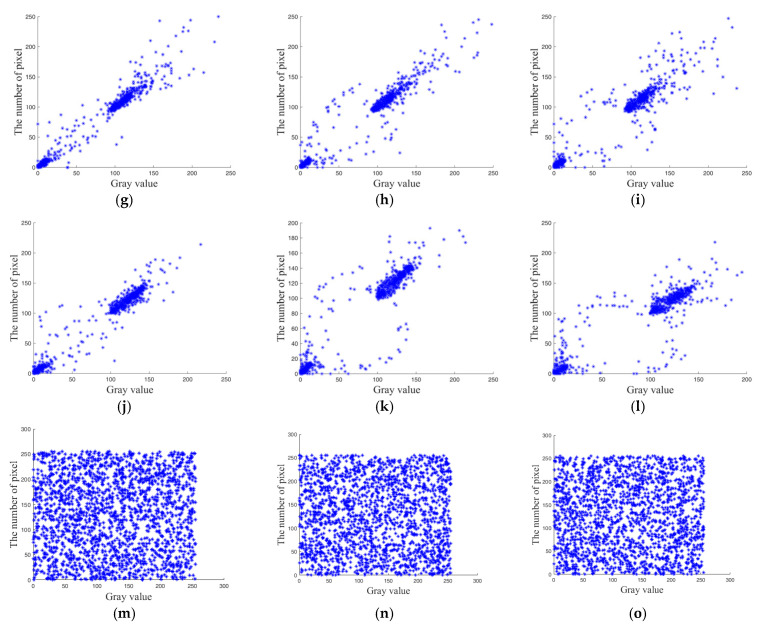
Correlation analysis. (**a**–**c**) Horizontal, vertical, and diagonal correlation of original “001” image. (**d**–**f**) Horizontal, vertical, and diagonal correlation of original “002” image. (**g**–**i**) Horizontal, vertical, and diagonal correlation of original “003” image. (**j**–**l**) Horizontal, vertical, and diagonal correlation of original “004” image. (**m**–**o**) Horizontal, vertical, and diagonal correlation of encrypted image.

We assessed the security of this approach by contrasting the correlation between neighboring pixels before and after encryption in order to examine how well it can withstand statistical analysis attacks. In order to achieve this, 2000 neighboring pixels were chosen at random from plain and cipher images in the horizontal, vertical, and diagonal directions. The pixel correlation coefficient can be calculated using Equation (20),and a scatter plot is given in Figure 7.

(20) $$r_{xy}=\frac{\mathrm{cov}(x,y)}{\sqrt{D(X)D(y)}},$$

(21) $$\mathrm{cov}(x,y)=\frac{1}{N}\sum_{i=1}^{N}\left(X_{i}-\frac{1}{N}\sum_{j=1}^{N}x_{i})(y_{i}-\frac{1}{N}\sum_{j=1}^{N}y_{i}\right),$$

(22) $$D(x)=\frac{1}{N}\sum_{i=1}^{N}{\left(x_{i}-\frac{1}{N}\sum_{j=1}^{N}x_{i}\right)}^{2},$$

where *x*, *y* are the values of two adjacent pixels, and $r_{xy}$ is the correlation coefficient of two adjacent pixels.

The correlation between adjacent pixels in plain images is very strong, as shown in the correlation distribution diagrams of adjacent pixels, and the distribution trend of pixel points is roughly linear or scattered, in contrast to the pixel point distribution of cipher images encrypted by this encryption algorithm, which covers the entire plane and shows that the correlation between adjacent pixels is significantly reduced after encryption by this algorithm.

Table 5 lists the correlation coefficients of plain images and cipher image in the horizontal, vertical, and diagonal directions. It can be seen that in plain images, the absolute value of the correlation coefficient is close to 1, indicating that the correlation coefficient is very high; in cipher images, the absolute value of the correlation coefficient is almost 0, indicating that adjacent pixels are almost uncorrelated. Table 6 shows the comparison of the correlation coefficients. It can be seen that the encryption algorithm proposed in this paper is more secure. 

### 4.3. Plaintext Sensitivity Analysis

In the plain image attack method, foreign attackers frequently alter the same plain image in different ways, such as changing a pixel’s value or position, to create a test image that differs only slightly from the original plain image and then create the corresponding cipher image using the same encryption algorithm. To attack the encryption algorithm, they compare the changes in the cipher image before and after the change and analyze the correlation between the two images. Such attacks are known as differential attacks. For the plain image that has only slightly changed during the encryption process, the encryption algorithm should generate a cipher image that is significantly different from the original cipher. This makes it difficult for the attacker to obtain the association between the plain image and the cipher image, preventing the encryption algorithm from being easily cracked by a differential attack.

Generally, the NPCR (number of pixels change rate, NPCR), which is the change rate of prime numbers, and the UACI (unified average change intensity, UACI), which is the normalized average change intensity, are used to test the resistance of cryptographic algorithms to differential attacks. NPCR represents the ratio of different gray values of different cipher images at the same location. The ideal value is 100%. The closer the result is to 100%, the greater the difference between cipher images generated by the encryption algorithm for the subtle changes in plain images. UACI represents the average change density between different cipher images. When the value is closer to 33.3333%, this indicates that the algorithm can better resist differential attacks.

Note that the plain images with only one pixel value different are $I_{1}$ and $I_{2}$, and the corresponding cipher images are $Q_{1}$ and $Q_{2}$. The NPCR and UACI values between the two cipher images can be calculated using Equations (23) and (24):

(23) $$NPCR=\frac{1}{M\times N}\sum_{i=1}^{M}\sum_{j=1}^{N}D(i,j)\times 100\%,$$

(24) $$UACI=\frac{1}{M\times N}\sum_{i=1}^{M}\sum_{j=1}^{N}\frac{\left|C_{1}(i,j)-C_{2}(i,j)\right|}{255}\times 100\%,$$

where the length and width of the image are represented by *M* and *N*, while $C_{1}(i,j)$ and $C_{2}(i,j)$ represent the pixel values of the (i,j) position on the cipher image before and after changing one pixel of the plain image, respectively. If $C_{1}(i,j)\neq C_{2}(i,j)$D(i,j)=1; otherwise, D(i,j)=0. We tested the image in Figure 2, and the results are shown in Table 7 and Table 8. It can be seen that the values of NPCR and UACI are close to 100% and 33.33%, respectively, indicating that this method has strong resistance to differential attacks.

### 4.4. Key Sensitivity Analysis

**Figure 8:**
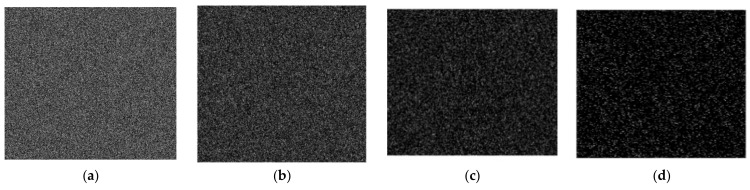
Key sensitivity test. (**a**) Decrypted “001” image with the wrong key, (**b**) decrypted “002” image with the wrong key, (**c**) decrypted “003” image images with the wrong key, (**d**) decrypted “004” image with the wrong key.

Because of the algorithm’s high key sensitivity, even a small change in the key used for decryption can have a significant impact on the outcomes of decrypting the same encrypted image. We randomly change one bit of the original encryption key before decrypting the cipher image to check the method’s key sensitivity. The outcomes are displayed in Figure 8. It is clear that the resultant decryption image cannot view any information of the plain image, even if the key simply changes by one bit.

### 4.5. Information Entropy Analysis

The most crucial concept in information theory is called information entropy, and it describes the numerous uncertain aspects of potential events in information sources. The degree of uniformity in a system’s state can be determined by the system’s entropy value. The system state is said to be ordered if the entropy value is minimal, and disordered if the entropy value is large. Information entropy is used to quantify the uniformity of information since the signal transmitted by a specific source is unpredictable and ambiguous. The formula for computing information entropy is displayed in Equation (25):

(25) $$H(x)=-\sum_{i=0}^{255}p(x_{i})log_{2}p\left(x_{i}\right)$$

where $p(x_{i})$ represents the probability of occurrence of pixel gray value *x*. If the information entropy H(x)=8, it indicates that the information is completely random, so the disorder of encrypted information is stronger. We calculated the information entropy of the medical image in Figure 2 and the ciphertext image of the proposed method; the results are shown in Table 9. It can be seen that the information entropy of the encrypted image is close to 8, indicating that the encrypted information has strong disorder. Table 10 provides a comparison of the proposed algorithm with existing approaches in terms of entropy. It is seen that all values are very close to the theoretical value, and the entropy of the proposed method is compared to several other methods.

### 4.6. Shear Resistance

**Figure 9:**
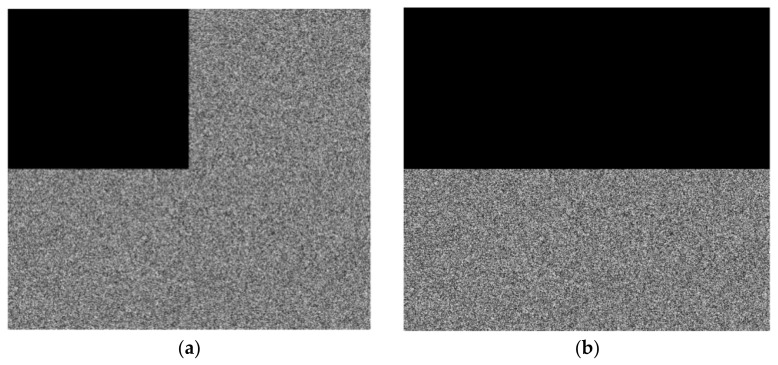
The example of cropping attack for an encrypted image. (**a**) Encrypted image with 25% data loss. (**b**) Encrypted image with 50% data loss.

**Figure 10:**
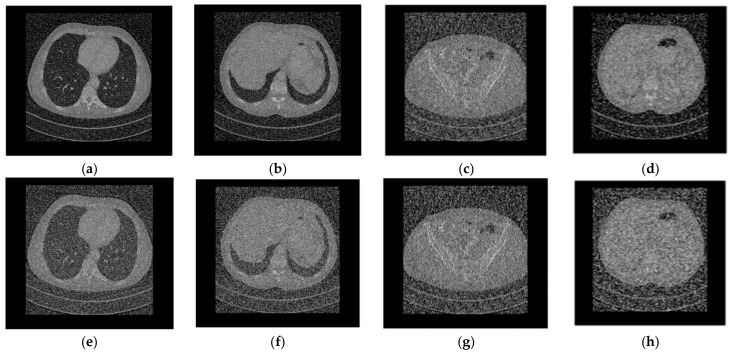
Decryption results of Figure 9. (**a**–**d**) Decrypted images of Figure 9a. (**e**–**h**) Decrypted images of Figure 9b.

During transmission and storage, images are impacted by a variety of variables, and occasionally, attackers may edit images to cause local loss. When the cipher image is attacked by cutting, a decent encryption method needs to be able to restore the key information of the plain image. To test the algorithm’s resistance to cutting, we cut the cipher image to remove some of its information and then used the same key to decrypt the modified cipher image. Figure 9 shows the 25% and 50% loss attacks on the encrypted image. Figure 10 shows the corresponding decryption results.

It is not difficult to see from the results that when 25% of the encrypted image is lost, the decrypted image is still very clear. With the increase in the cut area, the decrypted image begins to blur slowly. Even if 50% of the encrypted image is cut, the decrypted image still has the ability to express the original medical image information. In order to analyze the decrypted image quality of the cut encrypted image, we calculated the peak signal-to-noise ratio (PSNR) of the decrypted image in Figure 10. The results are shown in Table 11. The test results show that although the secret image is cut, it can still decrypt a more reliable decrypted image, which shows that the method has a good anti-cutting ability.

### 4.7. Anti-Noise Attack

**Figure 11:**
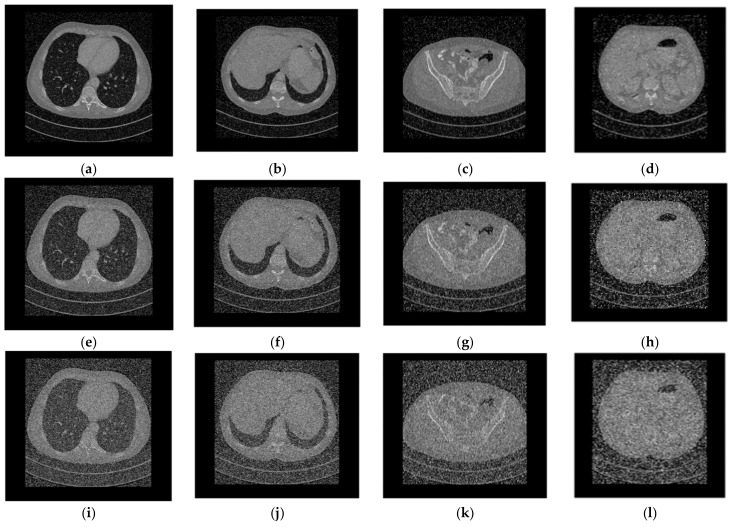
Decrypted images with different salt and pepper noise intensity. (**a**–**d**) Decrypted images after adding salt and pepper noise with 0.1 intensity to the ciphertext image. (**e**–**h**) Decrypted images after adding salt and pepper noise with 0.3 intensity to the ciphertext image. (**i**–**l**) Decrypted images after adding salt and pepper noise with 0.5 intensity to the ciphertext image.

Noise frequently interferes with information transmission, resulting in discrepancies between the received ciphertext and the actual ciphertext. A good image encryption technique must be anti-noise, meaning that the ciphertext can still decrypt the plain image’s key information despite being subjected to various noise attacks. To check the anti-noise attack capability of the technique, we added salt and pepper noise of different intensities to the ciphertext and decrypted the noisy cipher image using the same key. The experimental results are depicted in Figure 11.

As shown in Figure 11, as the noise intensity increases, the decrypted image becomes increasingly blurry, but the original image’s key information is still discernible, indicating that the algorithm has anti-noise capability. The peak signal-to-noise ratios of the corresponding decrypted image are given in Table 12.

### 4.8. Efficiency Analysis

Due to its high speed and parallelism, DNA computing has the advantage of encrypting large numbers of image data. Table 13 shows the time consumed for the encryption and decryption of medical images of different numbers and sizes. All test were performed using the software and hardware mentioned in Section 3. It can be seen that the greater the number of encryption images, the greater the advantage of the algorithm in terms of time efficiency.

## 5. Conclusions

Encrypting medical images is crucial for protecting patients’ privacy and personal information. This research presents a method for medical image encryption that can simultaneously encrypt any number of images of any size. It has the same computational cost as encrypting a single image. The decryption process is capable of performing single-image decryption, eliminating the possibility of information leaking. After testing and performance analysis, the encryption algorithm can achieve good encryption results, can resist various attacks, and has a high security level and fast encryption speed.

## Figures and Tables

**Table 1:** The rule of coding.

	1	2	3	4	5	6	7	8
A	11	10	01	01	00	10	00	11
C	01	00	00	11	10	11	01	10
G	10	11	11	00	01	00	10	01
T	00	01	10	10	11	01	11	00

**Table 2:** DNA addition.

+	A	C	G	T
A	A	C	G	T
C	C	G	T	A
G	G	T	A	C
T	T	A	C	G

**Table 3:** DNA subtraction.

−	A	C	G	T
A	A	T	G	C
C	C	A	T	G
G	G	C	A	T
T	T	G	C	A

**Table 4:** DNA XOR operation.

⊕	A	C	G	T
A	A	C	G	T
C	C	A	T	G
G	G	T	A	C
T	T	G	C	A

**Table 5:** The correlation coefficients of adjacent pixels.

Image	Horizontal	Vertical	Diagonal
“001”	0.9944	0.9902	0.9807
“002”	0.9948	0.9902	0.9859
“003”	0.9918	0.9880	0.9810
“004”	0.9907	0.9750	0.9639
ciphertext	−0.0164	0.0056	0.0289

**Table 6:** The comparison of correlation coefficients.

Algorithm	Horizontal	Vertical	Diagonal
Ref. [7]	0.9944	0.9902	0.9807
Ref. [9]	0.9948	0.9902	0.9859
Ref. [10]	0.9918	0.9880	0.9810
Ref. [12]	−0.0164	0.0056	0.0289

**Table 7:** The test results of NPCR and UACI.

Image	NPCR	UACI
Change a pixel in 001	99.6128	33.5196
Change a pixel in 002	99.6025	33.4625
Change a pixel in 003	99.5995	33.4191
Change a pixel in 004	99.6094	33.4616

**Table 8:** The comparison of average NPCR and UACI values.

Algorithm	NPCR	UACI
Proposed	99.6174	33.4657
Ref. [9]	99.6019	33.4538
Ref. [7]	99.6128	33.4640
Ref. [10]	99.6005	33.4399
Ref. [12]	99.6153	33.4353

**Table 9:** Information entropy results.

“001” Image	“002” Image	“003” Image	“004” Image	Encrypted Image
4.9309	4.8542	4.7429	4.8007	7.9993

**Table 10:** Comparison of the proposed algorithm with existing approaches in terms of entropy.

Algorithm	Ref. [7]	Ref. [9]	Ref. [10]	Ref. [12]	Proposed
Ciphertext (avg)	7.9985	7.9980	7.9990	7.9992	7.9993

**Table 11:** PSNR of the plain image obtained by decrypting the encrypted image with data loss.

PSNR	Encrypted Image with 25% Data Loss	Encrypted Image with 50% Data Loss
“001” Image	19.9670	18.4261
“002” Image	19.3306	17.7964
“003” Image	19.3469	17.7690
“004” Image	19.0786	17.5803

**Table 12:** PSNR of the plaintext by decrypting the ciphertext after the noise attack.

PSNR	Intensity 0.1	Intensity 0.3	Intensity 0.5
“001” Image	21.9331	19.5072	18.4124
“002” Image	21.2920	18.8837	17.7666
“003” Image	21.1739	18.8303	17.7299
“004” Image	21.0209	18.6305	17.5242

**Table 13:** The encryption and decryption time test(unit: seconds).

Number of Images	Encryption	Decryption
4	1.652	1.207
5	1.987	1.619
6	2.267	1.904
7	2.669	2.085
8	3.588	3.011

## Data Availability

Data is contained within the article.
